# Clinical, biochemical and molecular phenotype of congenital disorders of glycosylation: long-term follow-up

**DOI:** 10.1186/s13023-020-01657-5

**Published:** 2021-01-06

**Authors:** Anna Bogdańska, Patryk Lipiński, Paulina Szymańska-Rożek, Aleksandra Jezela-Stanek, Dariusz Rokicki, Piotr Socha, Anna Tylki-Szymańska

**Affiliations:** 1 Department of Biochemistry, Radioimmunology and Experimental Medicine, The Children’s Memorial Health Institute, Warsaw, Poland; 2 Department of Pediatrics, Nutrition and Metabolic Diseases, The Children’s Memorial Health Institute, Warsaw, Poland; 3 Faculty of Mathematics, Informatics and Mechanics, University of Warsaw, Warsaw, Poland; 4 Department of Genetics and Clinical Immunology, National Institute of Tuberculosis and Lung Diseases, Warsaw, Poland; 5 Department of Gastroenterology, Hepatology, Feeding Difficulties and Pediatrics, The Children’s Memorial Health Institute, Warsaw, Poland

**Keywords:** Glycosylation, Congenital disorders of glycosylation, Serum transferrin isoforms, Follow-up

## Abstract

**Background:**

Congenital disorders of glycosylation (CDG) result from defects in the synthesis of glycans and the attachment of glycans to proteins and lipids. Our study aimed to describe the clinical, biochemical, and molecular findings of CDG patients, and to present the long-term follow-up.

**Material and methods:**

A single-center study (1995–2019 years) of patients with congenital disorders of N-glycosylation and combined N- and O-hypoglycosylation was performed.

**Results:**

Among 32 patients included into the study, there were 12 PMM2-CDG, 3 ALG13-CDG, 3 ALG1-CDG, 1 ALG3-CDG, 3 MPI-CDG, 1 PGM1-CDG, 4 SRD5A3-CDG, 1 DPAGT1-CDG, 3 ATP6AP1-CDG, 1 ATP6V0A2-CDG. The phenotypic and genotypic spectrum during long-term (in some cases over 20 years) observation was characterised and several measurements of serum Tf isoforms taken. Statistical analysis revealed strong negative correlation between asialo-Tf and tetrasialo-Tf, as well as between disialo-Tf and tetrasialo-Tf. Within CDG type I, no difference in % Tf isoforms was revealed between PMM2-CDG and non-PMM2-CDG patients. However, these two groups differed significantly in such diagnostic features as: cerebellar ataxia, failure to thrive, hypothyroidism, pericardial effusion, cardiomyopathy, inverted nipples, prolonged INR.

The effect of treatment with mannose in 2 patients with MPI-CDG was assessed and we found that % of asialo-Tf, monosialo-Tf, and disialo-Tf was significantly lowered, whereas tetrasialo-Tf and pentasialo-Tf rose, coming closer or falling into the reference range.

**Conclusions:**

The novel finding was an abnormal Tf IEF pattern in two ALG13-CDG patients and normal in one ALG1-CDG patient. Clinical manifestation of presented CDG patients was similar to that reported in the literature. Mannose supplementation in MPI-CDG patients, as well as galactose supplementation in PGM1-CDG patient, improved patients’ clinical picture and Tf isoform profiles.

## Background

Congenital disorders of glycosylation (CDG), first reported in 1980, result from defects in the synthesis of glycans and the attachment of glycans to proteins and lipids [1]. According to the current nomenclature, CDG can be classified into defects in protein N-glycosylation, O-glycosylation, glycosphingolipid and glycosylphosphatidylinositol anchor glycosylation defects, and multiple glycosylation pathways defects. Since the description of phosphomannomutase 2 deficiency (PMM2-CDG), more than 130 other CDG subtypes have been reported [2, 3].

So far, single case reports and case series regarding various CDG have been published, but the data regarding follow-up are sparse in the literature. Our study aimed to describe the clinical, biochemical, including serum Tf isoform analysis, as well as molecular features of patients diagnosed with CDG in one referral center, and to present their long-term follow-up.

## Material and methods

**Fig. 1:**
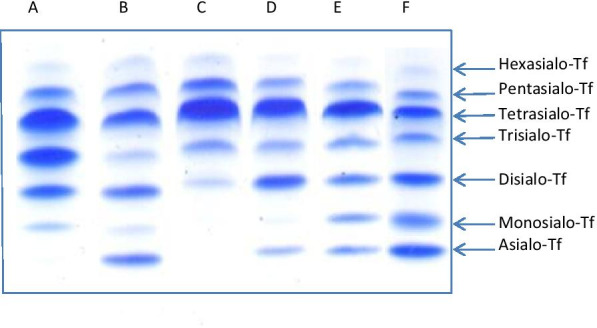
Isoelectrofocusing (IEF) of serum transferrin isoforms (**a** pattern for patient with CDG type II, elevated monosialo-, disialo- and trisialotransferrin fraction; **b** pattern for patient with CDG type I, elevated asialo- and disialotransferrin isoform; **c **control normal profile; **d** pattern for patient with mild CDG type I, slightly elevated asialo- and elevated disialotransferrin fraction; **e** pattern for patient with mixed CDG I/II on galactose supplementation, mild elevated asialo-, monosialo- and disialotransferrin isoforms; **f** pattern for patient with mixed CDG I/II before treatement, elevated asialo-, monosialo-, and disialotransferrin isoforms)

During 1995–2019 years, a total number of 22,063 serum Tf isoform analysis, have been done in our Institute. Serum Tf isoforms were analyzed by isoelectrofocusing (IEF) agarose gel electrophoresis according to our modification [4, 5] of the method described by Van Eijk et al. [6]. Serum Tf consists of the mixture of isoforms and has two iron binding sites, which was saturated with iron (20 μl serum with 80 μl 0,9% NaCl, 2 μl 10 mM Fe(III) citrate and 2 μl 0.1 M NaHCO_3_). Transferrin migrated through 1% agarose gel (45 mg of agarose were added to 4.5 ml of distilled water) with 5% ampholines in a pH range of 5.0–7.0 on a Multiphore 2117 apparatus (LKB) with a modified electrode lid. Tf isoforms were visualized by immunofixation (200 µl polyclonal rabbit anti-human transferrin serum) and 0.5% Coomasie Brillant Blue solution staining, as presented in Fig. 1. The percentage of Tf fractions was assessed densitometrically and carbohydrate deficient transferrin value (% CDT) was measured as a sum of asialo-, monosialo-, and disialo-Tf isoforms.

Serum O-glycoprotein apolipoprotein C-III (ApoC-III) isoforms were analyzed by isoelectrofocusing polyacrylamide gel electrophoresis according to the method described by Wopereis et al. [7]. The activity of phosphomannomutase 2 (PMM2; EC 5.4.2.8) and mannosephosphate isomerase (MPI; EC 5.3.1.8) in fibroblasts were determined according to the method of Van Schaftingen and Jaeken [8]. Phosphoglucomutase 1 (PGM1) activity was assayed spectrophotometrically on cell extracts [9, 10]. Lipid-linked oligosaccharide (LLO) profiles in fibroblasts were analyzed by high-performance liquid chromatography (HPLC) [11–13].

The molecular analysis using direct sequencing of single genes (*PMM2*, *MPI*, *PGM1*) was performed to confirm the proper biochemical diagnosis. In a group of patients with no diagnosis established based on abovementioned diagnostic procedures, whole-exome sequencing (WES) was applied. The nomenclature of identified variants and patients’ genotype follows the Human Genome Variation Society guidelines (HGVS v 2.0, www.hgvs.org/mutnomen) and referral according to cDNA and protein sequences of various CDG genes followed the Human Gene Mutation Database (HGMD, www.hgmd.cf.ac.uk).

Finally, 32 patients with molecularly confirmed congenital disorders of N-glycosylation and combined N- and O-hypoglycosylation, diagnosed and followed-up, have been recruited to the study.

The chart review of patients’ medical records concerning the demographics, first presented signs and symptoms, age at diagnosis, diagnostics methods (described below), clinical outcome, as well as biochemical (serum Tf isoforms, aspartate (AST) and alanine (ALT) aminotransferases, international normalized ratio (INR)), and molecular data were collected. Ethical approval was obtained from the Children’s Memorial Health Institute Bioethical Committee, Nr 23/KBE/2020, Warsaw, Poland.

An extended statistical analysis was performed. First, in order to find correlations between % of Tf isoforms, we calculated Pearson’s linear correlation coefficient r between all Tf isoforms. Next, we compared the subcohort of PMM2-CDG-I and non-PMM2-CDG-I patients in terms of the mean of % Tf isoforms. We also performed chi-squared test for independence to establish if diagnostic variables, such as muscle hypotonia, hypothyroidism, nystagmus, etc., are independent of the type of CDG-I disease. Finally, we investigated the effect of treatment administrated in three patients on the % Tf isoforms. We compared means (or single values) measured before treatment with the means of measurement taken after the first administration of mannose administration (in one patient with CDG-I/CDG-II) or galactose administration (two patients with non-PMM2-CDG-I).

The alpha level of significance was chosen 0.05. For computations we used statistical software “R”.

## Results

### Overall characteristics

Examining serum Tf isoform profile in our cohort of 32 patients, we established the following:

- 24 patients had type I Tf isoform profile (CDG-I),
- 4 patients had type II Tf isoform profile (CDG-II),
- 1 patient had a mixed type (CDG-I/CDG-II).

Out of 24 patients with CDG-I, PMM2 activity was found deficient in 12 patients, while MPI activity was found deficient in 3 patients. In one patient with CDG-I/CDG-II, PGM1 activity was found deficient. Molecular analysis using direct sequencing of single genes (*PMM2*, *MPI*, *PGM1*) confirmed the proper diagnosis.

In a group of patients with no diagnosis established based on abovementioned diagnostic procedures, whole-exome sequencing (WES) was applied. Thus, the diagnosis of ALG1-CDG, ALG3-CDG, DPAGT1-CDG, and SRD5A3-CDG was made.

Three patients were missed by serum Tf analysis. One of them (Patient 18) was diagnosed based on array comparative genomic hybridization in which chromosome 16p13.3 deletion involving *ALG1* gene was found. Three patients were diagnosed by WES with ALG13-CDG. Serum Tf isoform profile was normal in one of them (Patient 14), in one other (Patient 13) was indicative for CDG-I, and in the third one (Patient 15) disialo-Tf isoform was slightly elevated.

Four patients, including three of them from one family (Patients 30–32), with CDG-II showed an alteration in the apoC-III isoform profile (increased apoCIII-1, decreased apoCIII-2), indicative of a combined N- and O-glycosylation defect. ATP6AP1-CDG and ATP6V0A2-CDG were diagnosed by WES, respectively.

Our cohort of 32 patients breaked down to the following subgroups:

- 12 patients with PMM2-CDG,
- 3 patients with ALG13-CDG,
- 3 patients with ALG1-CDG,
- 1 patient with ALG3-CDG,
- 3 patients with MPI-CDG,
- 1 patient with PGM1-CDG,
- 4 patients with SRD5A3-CDG,
- 1 patient with DPAGT1-CDG,
- 3 patients with ATP6AP1-CDG,
- 1 patient with ATP6V0A2-CDG.

Some of the patients have been previously reported as single case reports or case studies [10, 14, 15]. Detailed characteristic is presented in Additional file 2: Supplementary Table S1.

To further analyses, patients were divided into two groups:

- 12 patients with PMM2-CDG and
- 20 with non-PMM2-CDG.

### PMM2-CDG patients

Twelve patients with PMM2-CDG were identified. All of them had an early-onset (within 12 months of age) of the disease. Non-immune hydrops foetalis (NIHF) was present in two of them.

#### Presentation at diagnosis

The frequency of observed symptoms and features in the cohort of PMM2-CDG patients is given in Table 1. It is compared to the frequency of symptoms in the cohort of non-PMM2-CDG.

**Table 1:** Number of patients presenting a given symptom in the two subcohorts of PMM2-CDG patients and non-PMM2-CDG patients. P-value of the chi-squared test for independence of variables “feature/type of CDG” given in last column; symptoms for which the p-value was less than 0.05 marked with colour

Feature	PMM2-CDG	Non-PMM2-CDG	*p* value of the chi-squared test for independence
Number of patients	12	20	
Muscle hipotonia	10/12	9/20	0.08
Motor retardation	12/12	13/20	0.06
Cerebellar ataxia	10/10	4/20	0.0002
Seizures	3/12	5/20	1
Microcephaly	2/12	6/20	0.67
Visual impairment	8/12	6/20	0.1
Strabismus	4/12	1/20	0.1
Nystagmus	1/12	4/20	0.71
Optic nerve hypoplasia/atrophy	0/12	1/20	1
Recurrent vomiting/diarrhea	0/12	1/20	1
Hepatomegaly	8/12	6/20	0.1
Failure to thrive	9/12	6/20	0.04
Hypothyroidism	8/10	0/20	< 0.0001
Proteinuria	2/12	2/20	1
Pericardial effusion	6/12	1/20	0.01
Cardiomyopathy	5/12	1/20	0.04
Inverted nipples	8/12	0/20	0.0001
Cutis laxa	0/12	1/20	1
Prolonged INR	6/12	2/20	0.04
Elevated serum transaminases	8/12	13/20	1
Low antithrombin III, protein C, and S	6/9	12/15	0.81

In PMM2-CDG patients, 13 various pathogenic variants in the *PMM2* gene (NM_000303.2) have been identified, including 10 missense mutations, 2 intronic mutations and 1 frame-shift mutation. The most frequent mutations were c.691G > A, p.Val231Met (22%) and c.422G > A, p.Arg141His (22%).

#### Serum Tf IEF

**Fig. 2:**
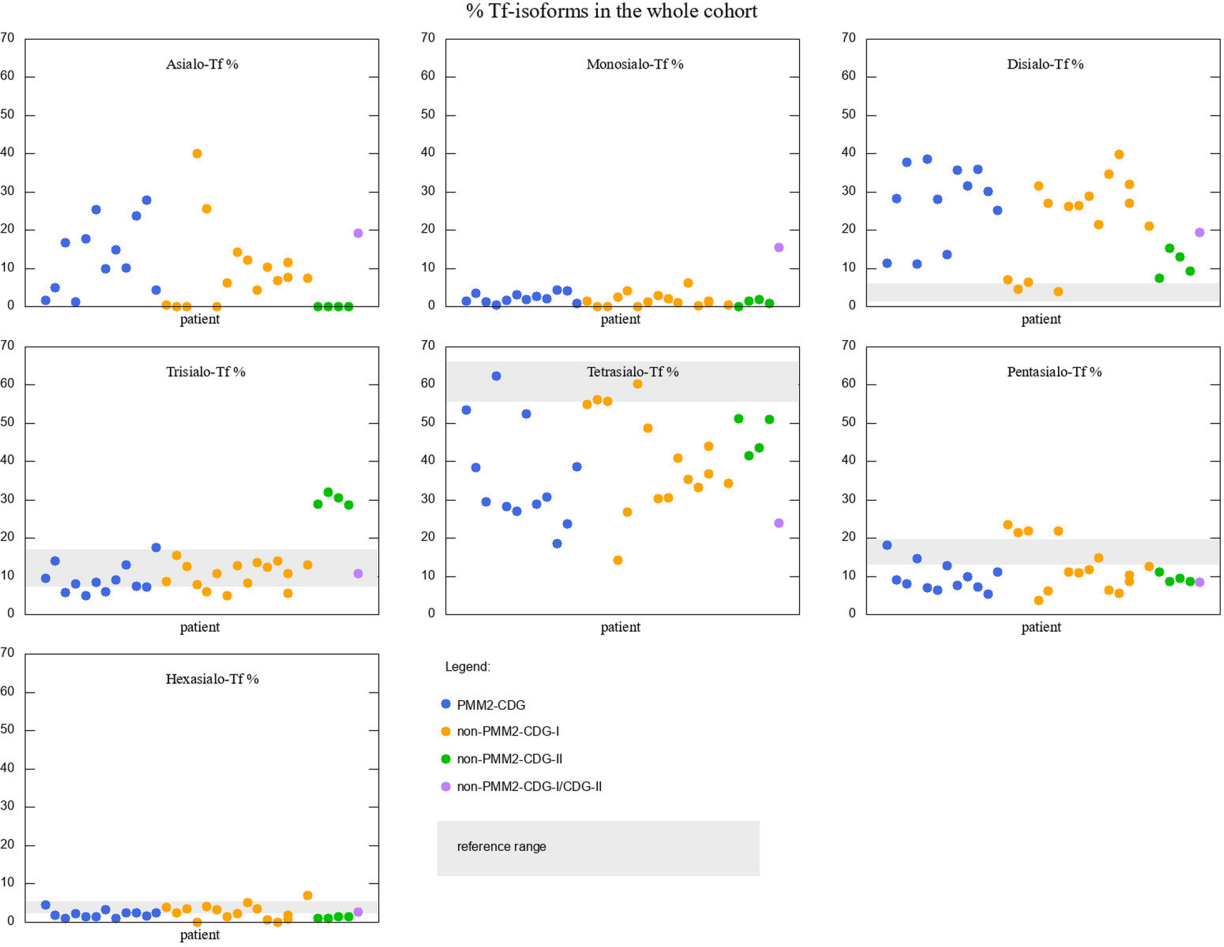
Tf isoforms percentage for the whole cohort of patients, different types of CDG marked with different colours. Grey shades represent the reference ranges. For asialo-Tf and monosialo-Tf the reference value is 0

Serum Tf IEF in all patients showed elevated asialo- and disialo-Tf isoforms. Eleven out of twelve PMM2-CDG patients had low tetrasialo-Tf. Levels of all seven Tf isoforms of patients with PMM2-CDG are presented in Fig. 2 (first twelve dots, blue).

#### Follow-up

Eleven out of twelve patients were followed-up. The mean time of follow-up was 8 years (range 8 months–18 years). At the last follow-up, 10 patients were alive while two others (Patient 6 and 7) had died at the age of 9 and 2 months, respectively. Patient 6 demonstrated prenatally NIHF; the exact cause of death is not known. Patient 7 had a neurovisceral form of the disease with liver and cardiac involvement, and was on mechanical ventilation since the second week of life. *Post mortem* examination revealed liver cirrhosis.

Improvement of motor skills was observed in the majority of patients except for three alive patients with cardiac features. Due to massive pericardial effusion, two of them required pericardiocentesis, and pleural-pericardial window formation. In two, feeding by a nasogastric tube was needed.

### Non-PMM2-CDG patients

There were 20 patients, including three ALG13-CDG, three ALG1-CDG, one ALG3-CDG, three MPI-CDG, one PGM1-CDG, four SRD5A3-CDG, one DPAGT1-CDG, three ATP6AP1-CDG, and one ATP6V0A2-CDG.

All the patients presented an early-onset (within 2 years of age) disease. One patient diagnosed with ATP6AP1-CDG deficiency had NIHF.

#### Presentation at diagnosis

The frequency of observed symptoms and features in the cohort of non-PMM2-CDG patients is given in Table 1.

Among 15 patients, 12 various pathogenic variants in the CDG associated genes have been identified, including 11 missense mutations and 1 frame-shift mutation. All of them were previously reported.

#### Serum Tf IEF

Serum Tf IEF was abnormal in 18 out of 20 patients. One of the patients with ALG13-CDG and one with ALG1-CDG had normal Tf IEF while other patients with ALG1-CDG had significantly increased asialo- and disialo-Tf. Tf isoform profile showed slightly elevated asialo- and disialo-Tf in the first patient with ALG13-CDG and slightly elevated disialo-Tf in the third patient with ALG13-CDG. All patients with MPI-CDG presented a decrease in percentage of CDT on mannose supplementation. Four patients with SRD5A3-CDG showed similar Tf isoform values and %CDT within 40.1–47.1. Patient with DPAGT1-CDG had a typical type I Tf isoform profile with %CDT 33.2.

Four patients had type II Tf isoform profile: three with ATP6AP1-CDG, and one with ATP6V0A2-CDG. ATP6V0A2-CDG patient showed mildly elevated disialo-, elevated trisialo-Tf, and decreased tetrasialo-Tf. Patients with ATP6AP1-CDG had elevated disialo- and trisialo-Tf. Tertrasialo-Tf was decreased in all four patients. Levels of all seven Tf isoforms of patients with non-PMM2-CDG are presented in Fig. 2. Yellow dots represent non-PMM2-CDG-I patients and green dots represent non-PMM2-CDG-II patients.

PGM1-CDG patient showed a mixed type I/II Tf isoform profile with significantly increased asialo-, monosialo- and disialo-Tf, and highly decreased tetrasialo-Tf. Tf isoforms of this patient are depicted with purple dots in Fig. 2. The improvement of Tf isoform on galactose supplementation was noted. Measurements before the start of treatment and after the start of treatment with respective means are given in Fig. 4.

#### Follow-up

Among 20 patients, 19 are followed-up. The mean time of follow-up was 7 years (range: 1 month–19 years). At the last follow-up, 15 patients were alive while 4 others had died (two ALG1-CDG, one PGM1-CDG, one DPAGT1-CDG).

Patients with ALG1-CDG presented a severe phenotype leading to death in the first months of life.

MPI-CDG patients demonstrated the improvement of clinical and biochemical features on mannose supplementation.

Patient with PGM1-CDG manifested progressive cardiac insufficiency and liver impairment (hepatomegaly, elevated serum transaminases) since 4 years of age. Diagnosis of PGM1-CDG was established at 10 years of age, the galactose supplementation was started at the age of 16 years. The patient died being 19 years old due to cardiac failure.

Three out of four patients with SRD5A3-CDG showed an improvement of motor skills and speech development, the other one (Patient 26) demonstrated spastic tetraparesis and needed gastrostomy insertion.

The progressive disease course was observed in three males affected with ATP6AP1-CDG. Sensorineural hearing loss up to total deafness, as well as progressive hair loss up to total alopecia was observed in all of them. Two of them (Patient 30 and 31) also developed glomerular proteinuria.

### Statistical analysis

#### Correlations between isoforms

**Fig. 3:**
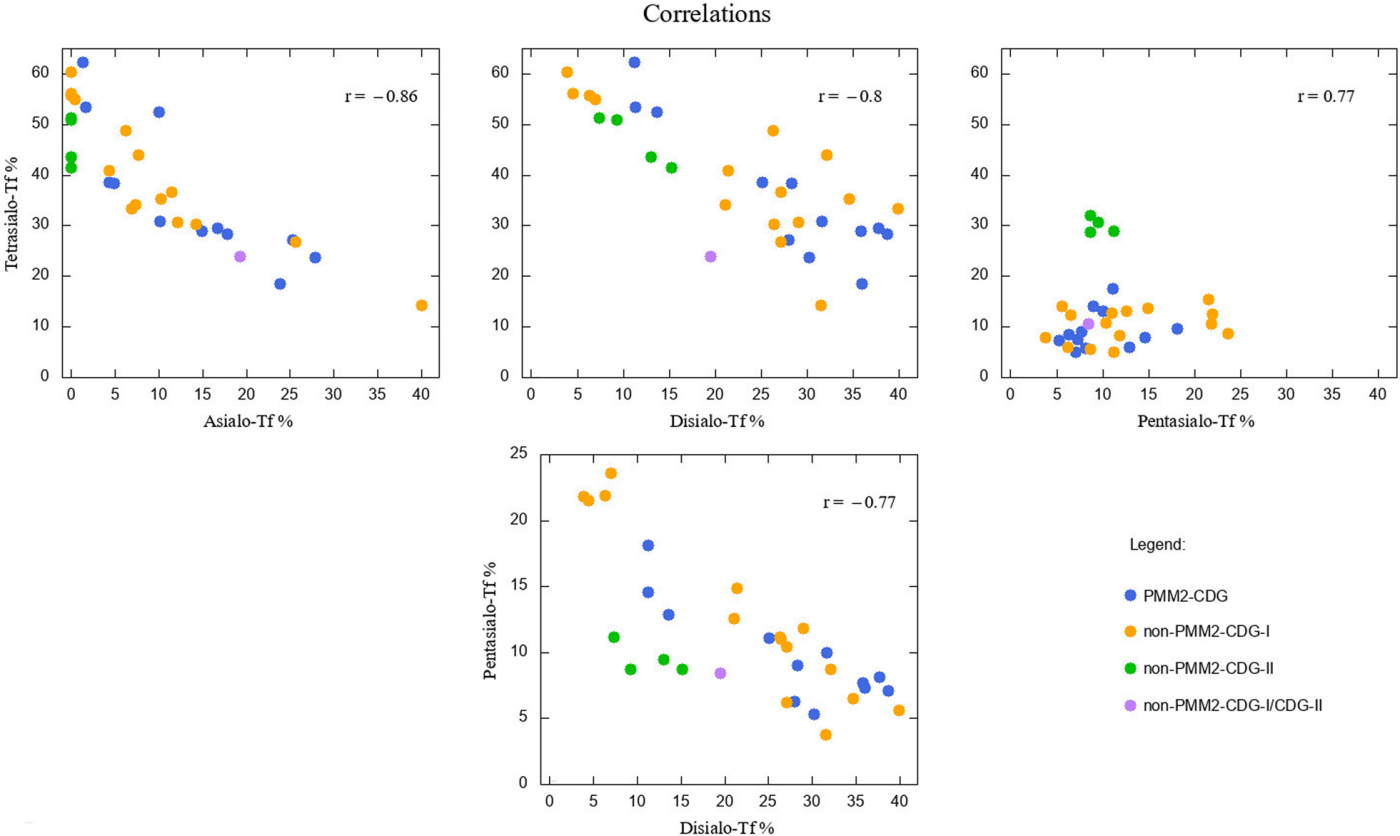
Correlations between chosen Tf isoforms % in the whole cohort of patients. Pearsons linear correlation coefficients r given in the upper right corner of each panel. Different colours used for different CDG variants

We calculated Pearson’s linear correlation coefficients r between all Tf isoforms, finding that the most strong, negative correlations exists between asialo-Tf and tetrasialo-Tf (r = − 0.86), disialo-Tf and tetrasialo-Tf (r = − 0.8), disialo-Tf and pentasialo-Tf (r = − 0.77). The only strong and positive correlation was revealed between tetrasialo-Tf and pentasialo-Tf (r = 0.78). These results differ only slightly between the subcohort of PMM2-CDG and non-PMM2-CDG patients. Detailed results are given in Additional file 3: Supplementary Table S2. Four strongest correlations between Tf isoforms are depicted in Fig. 3.

#### Comparison of PMM2-CDG and non-PMM2-CDG

We performed t-test (or its variant—Welsh test, where appropriate) to establish the statistical significance of the differences in means of Tf isoforms between PMM2-CDG and non-PMM2-CDG. We found that none of the Tf isoform differ significantly between these two groups of patients. The means of % Tf isoforms and respective p-values are given in Additional file 4: Supplementary Table S3.

PMM2-CDG and non-PMM2-CDG patients were also compared in terms of presented symptoms. Using chi-squared test for independence, we found that the variable “PMM2-CDG or non-PMM2-CDG” is dependent of the following diagnostic variables: cerebellar ataxia, failure to thrive, hypothyroidism, pericardial effusion, cardiomyopathy, inverted nipples, prolonged INR. The whole set of analysed symptoms with the p-values of the chi-squared tests are given in Table 1.

#### Assessment of treatment

There were three patients for whom data on % Tf isoforms were available before and during treatment. One MPI-CDG patient had one measurement taken before the start of treatment and eleven during the treatment. We compared the mean of the results obtained during treatment with the value measured before the start of treatment using one-sample t-test. All % Tf isoforms changed (except for trisialo-Tf), bringing the values closer to the reference range. Asialo-, monosialo- and disialo-Tf dropped significantly (more than or approximately half), whereas tetrasialo- and pentasialo-Tf rose significantly and met their respective reference ranges.

The second MPI-CDG patient had four measurements taken before the start of treatment and twelve taken during the treatment. Similarly, asialo-, monosialo-, disialo-, and trisialo-Tf % dropped, whereas tetrasialo- and pentasialo-Tf % rose.

The PGM1-CDG patient had seven measurements performed before the start of treatment, and eleven on treatment. Here we performed t-test to compare the means before and during treatment, finding similar results to those in MPI-CDG patients: asialo-, monosialo-, and diasialo-Tf were lowered, whereas tetra- and pentasialo-Tf rose enough to fall into the reference range.

Results for all three patients, along with reference ranges for comparison, and p-values are presented in Additional file 5: Supplementary Table S4.

**Fig. 4:**
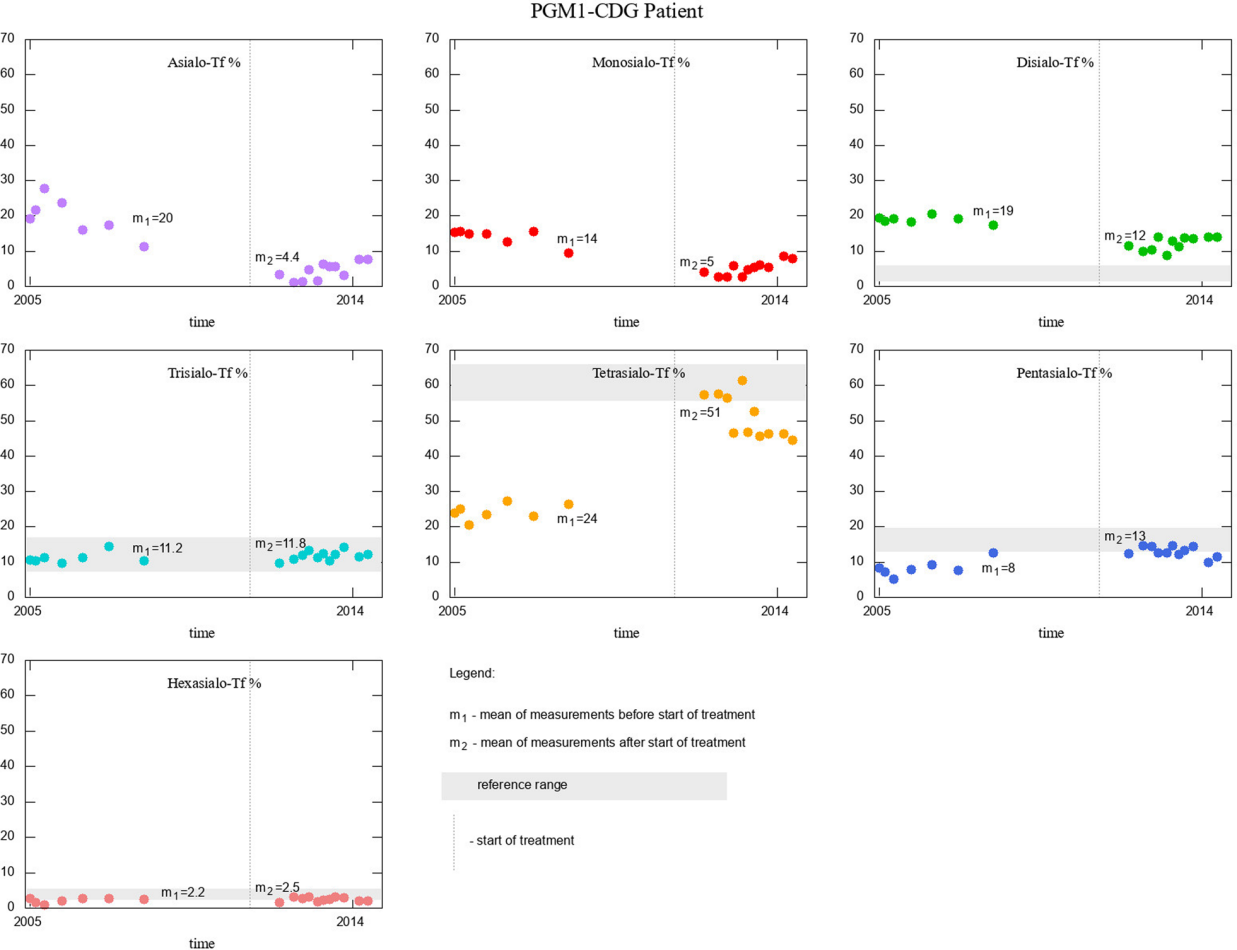
Effect of treatment in PGM1-CDG patient, for whom several measurements of Tf isoforms were available for the period before the start of treatment, and after. Vertical grey line represents the start of treatment, means of Tf isoforms % are given in each panel as m_1_ (before) and m_2_ (after the start of treatment)

We also depict the effect of galactose supplementation in PGM1-CDG patient in Fig. 4. An analogous visualisation for patients with MPI-CDG who had four measurements of % Tf isoforms taken before the start of mannose supplementation is given in Additional file 1: Supplementary Figure S1.

## Discussion

The paper presents the phenotypic and genotypic spectrum of 32 CDG patients during a long-term (over 20 years) observation in one center.

PMM2-CDG was the most common (40%) CDG identified in our study, similarly to the literature [16, 17]. Among the N-hypoglycosylation disorders, ALG6-CDG is reported as the second, while ALG1-CDG as the third most frequent type in the literature [18]. We did not identify any ALG6-CDG patients, whereas SRD5A3-CDG was the second most frequent type. ALG1-CDG, ALG13-CDG and MPI-CDG were comparably frequent.

Prenatal presentation (NIHF) was observed in three (9%) of our patients, including two PMM2-CDG (one died at two months of age). In a recent review by Makhamreh et al. (2020), the most common CDG associated with NIHF were PMM2-CDG, ALG9-CDG, and ALG8-CDG, which follows the line of our results [19]. Our paper is one of the first to report NIHF associated with ATP6AP1-CDG. Recently, thickened nuchal translucency and large fluid filled space with septations along the foetal spine have been described during the first trimester ultrasound in ATP6AP1-CDG foetus [20].

CDG patients frequently present neurological involvement [21]. The main neurological symptom in our patients remained psychomotor retardation and cerebellar ataxia with cerebellar hypoplasia on brain MRI scans, observed in 83% of PMM2-CDG patients and all SRD5A3-CDG patients. This finding confirms the thesis that the cerebellum is regularly involved in PMM2-CDG and SRD5A3-CDG [14, 25–27]. Epilepsy is also a common symptom (almost one-third of patients with PMM2-CDG in the study by Monin et al. [22]), in our cohort it was observed in ALG13-CDG patients. There are others CDG in which epilepsy is severe and difficult to control, like DPM1-CDG, DPM2-CDG, MPDU1-CDG, ALG2-CDG, ALG12-CDG, ALG8-CDG, ALG9-CDG, ALG11-CDG, and RTF1-CDG [23, 24].

Liver involvement was present in about 22% of all CDG in the last review by Marques-da-Silva et al. (2018) [28]. In our cohort, the predominant liver phenotype was observed in MPI-CDG patients, while other CDG like PMM2-CDG, ATP6AP1-CDG were associated with liver disease. Up to now, 14 patients have been reported with X-linked ATP6AP1 deficiency and the key features were immunodeficiency and liver involvement ranging from a mild elevation of serum transaminases to liver failure [15, 29, 30].

Our results are in agreement with the thesis that selected CDG have unique characteristics, which may facilitate and shorten their recognition. These include: connective tissue involvement in ATP6AP1-CDG, midline malformations in PGM1-CDG, chronic diarrhoea in MPI-CDG, inverted nipples and abnormal fat distribution in PMM2-CDG, cataract/coloboma in SRD5A3-CDG, cerebellar hypoplasia in PMM2-CDG and SRD5A3-CDG [31, 32]. Moreover, some of CDG are noted to manifest specific craniofacial dysmorphy which, if consistent with the entire clinical picture, allows for verification of the diagnosis. It refers especially to PMM2-CDG, manifesting with microcephaly, prominent forehead, flat nasal bridge, thin upper lip and large ears [31]. Recognizable facial features have been described in a number of other CDG [33, 34].

The clinical outcome of PMM2-CDG varies among patients [25]. Inverted nipples and abnormal fat distribution were reported as its characteristic features and were present in the majority of our patients. Pericardial effusion was noted in about 30% of PMM2-CDG patients [35]; in our cohort 50% of PMM2-CDG patients. Cardiac involvement in PMM2-CDG seems to be clinically relevant [35]; in our group five PMM2-CDG patients had cardiomyopathy, while it was a rare symptom in non-PMM2-CDG patients.

Abnormal thyroid function was reported in approximately 75% of PMM2-CDG patients [34]; we observed it with a comparable frequency. It results from an abnormal glycosylation of thyreotropine and thyroid-binding globulin [36].

Renal involvement was presented in 17% of PMM2-CDG patients from our cohort vs 6% of PMM2-CDG patients reported in the literature [36]. Our patients had proteinuria, and one of them congenital nephrotic syndrome. Like in the literature, mild proteinuria is the most common renal abnormality in those patients [37].

Some CDG have peculiar phenotypes being a combination of features. In our cohort, MPI-CDG manifested as purely hepatic (hepatomegaly, elevated liver transaminases) or hepato-intestinal (symptoms as above with recurrent diarrhoea) disease. In SRD5A3-CDG patients, cerebellar ataxia and signs of visual impairment and variable eye malformations, including optic disc hypoplasia, nystagmus, were the characteristic features. ATP6V0A2-CDG, as well as COG7-CDG, were described as cutis laxa syndrome with defective protein glycosylation [30, 38]. About 50% of patients with ATP6V0A2-CDG showed cortical malformations, especially pachygyria, in brain MRI. Our patient with ATP6V0A2-CDG showed cutis laxa, pachygyria, as well as polymicrogyria, which expands the spectrum of brain MRI features.

Isoelectric focusing (IEF) of serum transferrin (Tf) is still the method of choice for the diagnosis of N-glycosylation disorders associated with sialic acid deficiency. Our study showed for the first time that ALG13-CDG Tf IEF was abnormal in two our patients, while in ALG1-CDG one patient had normal Tf IEF. There were no reports on that in the literature. In every patient with clinical and biochemical diagnosis of CDG, molecular analysis has to be performed. It is essential to confirm the diagnosis and also to predict the possible genotype–phenotype correlation. In our study, WES analysis enabled the diagnosis of ALG13-CDG in patients with early-onset drug-resistant epileptic encephalopathy.

The most common pathogenic variants in the *PMM2* gene were c.691G > A, p.Val231Met and c.422G > A, p.Arg141His. The latter is also the most commonly reported in the literature [39]. The compound heterozygotes for p.Arg141H is noted in our study, and in the literature as well, were associated with a mild phenotype.

Two deceased patients with ALG1-CDG were compound heterozygous for the c.773C > T, p.Ser258Leu. This variant, if present in the homozygous state, is regarded to be related with an early fatal outcome.

Three out of four patients with SRD5A3-CDG presented with an improvement of motor skills and speech development, despite genetic alteration known to results in poor prognosis -c.292_293del, p.Leu98ValfsX121 (in two of them).

The data regarding follow-up of CDG is sparse in the literature. Our PMM2-CDG and SRD5A3-CDG patients showed an improvement in motor skills as well as no clinical progression of cerebellar symptoms. In ATP6AP1-CDG some symptoms aggravated with age (from sensorineural hearing loss to total deafness, from hair loss to total alopecia). ALG1-CDG forecast rather a poor outcome with death in the first months of life. Patients with MPI-CDG on mannose supplementation improved clinically and their Tf isoforms levels reached values close to the reference range. Patient with PGM1-CDG on galactose supplementation had died despite the improvement of TF isoforms.

## Conclusions

1. The novel finding was an abnormal serum Tf IEF pattern in two ALG13-CDG patients and normal in one ALG1-CDG patient.
2. Clinical manifestation of the presented cohort of CDG patients was similar to that reported in the literature.
3. MPI-CDG patients on mannose supplementation improved clinically and now function normally. Their Tf isoforms reached values close to the reference range.
4. PGM1-CDG patient on galactose supplementation had died despite the improvement of Tf isoforms.
5. Strong, negative linear correlations between asialo-Tf and tetrasialo-Tf, disialo-Tf and tetrasialo-Tf, disialo-Tf and pentasialo-Tf isoforms were detected. Strong, positive linear correlation was found between pentasialo-Tf and tetrasialo-Tf isoforms.
6. PMM2-CDG and non-PMM2-CDG differ significantly in the frequencies of the following symptoms: cerebral ataxia, failure to thrive, hypothyroidism, pericardial effusion, cardiomyopathy, inverted nipples, prolonged INR.

## Supplementary Information

**Additional file 1.** **Supplementary Figure S1.** Effect of treatment in MPI-CDG patient, for whom several measurements of Tf isoforms were available for the period before the start of treatment, and after. Vertical grey line represents the start of treatment, means of Tf isoforms % are given in each panel as m1 (before) and m2 (after the start of treatment).

**Additional file 2.** **Supplementary Table S1.** Detailed characteristics of study patients.

**Additional file 3.** **Supplementary Table S2.** Pearson’s correlation coefficients for % isoforms for the whole cohort of patients (A), PMM2-CDG patients (B), and non-PMM2-CDG patients (C). Correlation values |r|>0.7 marked with colour, the intensity of the hue reflecting the strength of the correlation.

**Additional file 4.** **Supplementary Table S3.** Comparison of means (with standard deviation) of Tf isoform % for the whole cohort of patients, PMM2-CDG patients and non-PMM2-CDG patients. The last column reports the p-values of the tests comparing the sample means (t-test or Welsh test).

**Additional file 5.** **Supplementary Table S4.** Means of % tf isoforms before and during treatment for CDG I/II patient (table A), patient #20 with MPI-CDG-I (table B) and patient #21 with MPI-CDG-I (table C). Comparison of means (A and B) or of mean with a single value (C) was performed with applicable statistical test (either t-test, Welsh test, or one-sample t-test).

## Data Availability

All data generated or analysed during this study are included in this published article.

## Abbreviations

ALT: Alanine aminotransferase
ApoC-III: Apolipoprotein C-III
AST: Aspartate aminotransferase
CDG: Congenital disorders of glycosylation
IEF: Isoelectrofocusing
LLO: Lipid-linked oligosaccharide
INR: International normalized ratio
MPI: Mannosephosphate isomerase
NGS: Next-generation sequencing
NIHF: Non-immune hydrops foetalis
PGM1: Phosphoglucomutase 1
PMM2: Phosphomannomutase 2
Tf: Serum transferrin
WES: Whole-exome sequencing
