# Supplementary figures and images for: Clinical, biochemical and molecular phenotype of congenital disorders of glycosylation: long-term follow-up
Source: Orphanet J Rare Dis. 2021 Jan 6;16:17. doi: 10.1186/s13023-020-01657-5 (PMC7789416; doi:10.1186/s13023-020-01657-5)

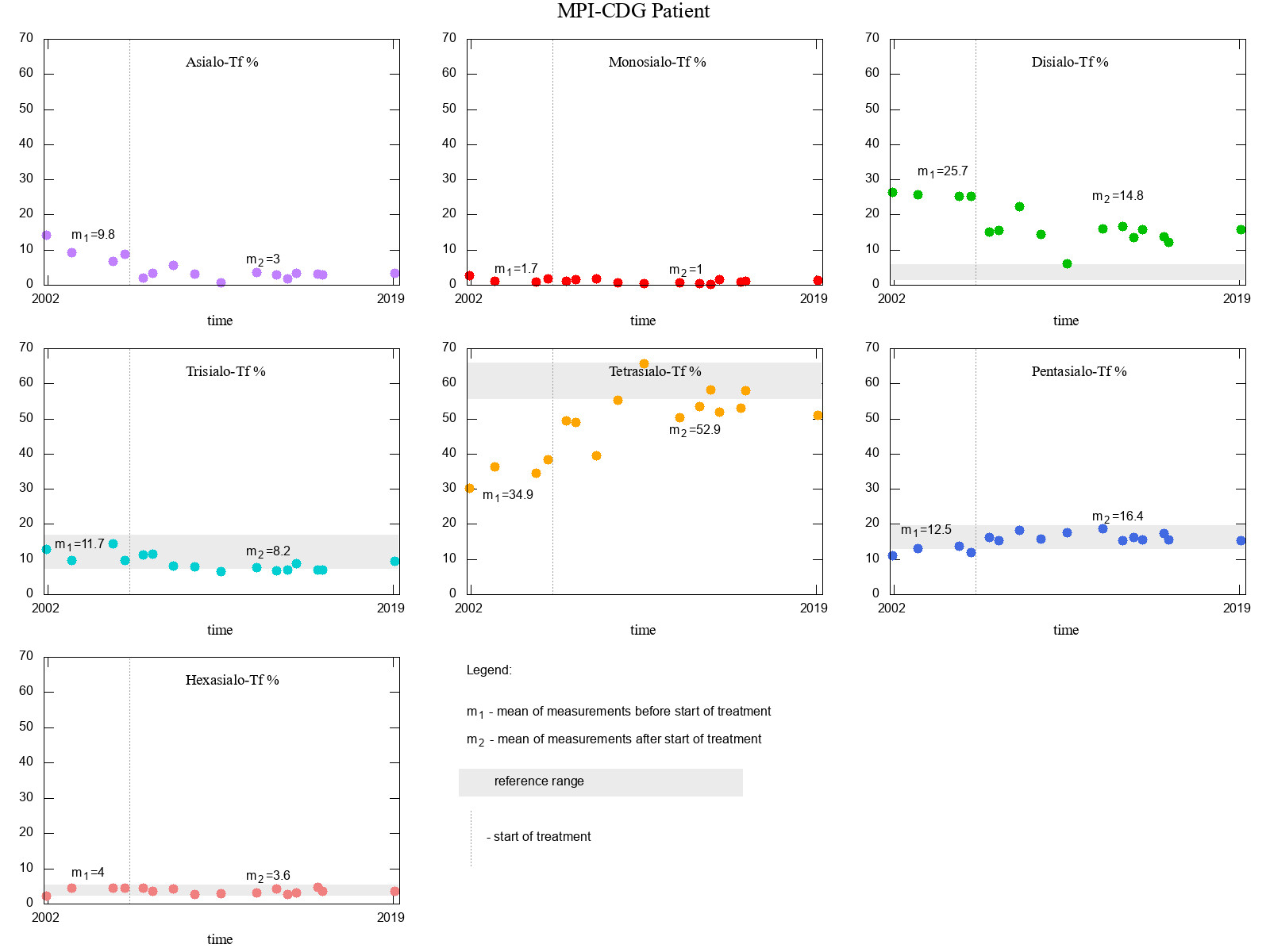

Supplement: Supplementary file 1 — Additional file 1. Supplementary Figure S1. Effect of treatment in MPI-CDG patient, for whom several measurements of Tf isoforms were available for the period before the start of treatment, and after. Vertical grey line represents the start of treatment, means of Tf isoforms % are given in each panel as m1 (before) and m2 (after the start of treatment). [file 13023_2020_1657_MOESM1_ESM.jpg]
