# Supplementary material for: Clinical, biochemical and molecular phenotype of congenital disorders of glycosylation: long-term follow-up
Source: Orphanet J Rare Dis. 2021 Jan 6;16:17. doi: 10.1186/s13023-020-01657-5 (PMC7789416; doi:10.1186/s13023-020-01657-5)
Supplement: Supplementary file 2 — Additional file 2. Supplementary Table S1. Detailed characteristics of study patients. [file 13023_2020_1657_MOESM2_ESM.doc]

| Patient | 1 | 2 | 3 | 4 | 5 | 6 | 7 | 8 | 9 | 10 | 11 | 12 |
| --- | --- | --- | --- | --- | --- | --- | --- | --- | --- | --- | --- | --- |
| Gender | M | M | M | M | M | M | F | F | M | M | F | M |
| Age of onset | 6m | 3m | 2m | Neonatal period | 4m | Prenatally (non-immune hydrops fetalis) | Neonatal period | Neonatal period | Neonatal period | Prenatally (non-immune hydrops fetalis) | Neonatal period | Neonatal period |
| Age at diagnosis | 6m | 3m | 5m | 6m | 6m | 1m | 1m | 6m | 2m | 2m | 2m | 1m |
| Diagnosis | PMM2 | PMM2 | PMM2 | PMM2 | PMM2 | PMM2 | PMM2 | PMM2 | PMM2 | PMM2 | PMM2 |  |
| Molecular analysis | c.155T>G,  p.V52G/  c.640-23A>G,  p? | c.422G>A,  p.R141H/  c.691G>A,  p.V231M | c.169G>A,  p.G57R/  c.422G>A,  p.R141H | c.422G>A,  p.R141H/  c.484C>T,  p.R162W | c.357C>A, p.F119L/ c.422G>A p.R141H | c.24delC,  p.C9AfsX27/  c.385G>A,  p.V129M | c.24delC,  p.C9AfsX27/  c.691G>A,  p.V231M | n.a. | c.691G>A,  p.V231M/  c.640-15479C>T | c.422G>A,  p.R141H/  c.691G>A,  p.V231M | c.710C>G,  p.T237R/  c.691G>A,  p.V231M | c.338C>T,  p.P113L;  c.470T>C,  p.F157S |
| Age at last follow-up | 8y | 16y | 4y5m | 17y | 6y | 9m | 2m | n.a. | 12y | 2y | 9m | 18y |
| Outcome | Alive | Alive | Alive | Alive | Alive | Died | Died | n.a. | Alive | Alive | Alive | Alive |
| **Presentation at diagnosis** | | | | | | | | | | | |  |
| **Neurological** |  |  |  |  |  |  |  |  |  |  |  |  |
| Muscle hipotonia | + | + | + | + | – | + | – | + | + | + | + | + |
| Motor retardation | + | + | + | + | + | + | + | + | + | + | + | + |
| Cerebellar ataxia with cerebellar hypoplasia in brain MRI | + | + | n.a. | + | + | + | + | n.a. | + | + | + | + |
| Seizures | + | + | – | – | – | – | – | – | + | – | – | – |
| Microcephaly | – | – | + | – | – | – | – | – | + | – | – | – |
| **Hearing impairment** | – | + | – | – | – | – | – | – | + | – | – | – |
| **Ocular manifestations** |  |  |  |  |  |  |  |  |  |  |  |  |
| Visual impairment | + | + | + |  | + | + | + | – | – | + | – | + |
| Strabismus | – | – | + | + | + | – | + | – | – | – | – | – |
| Nystagmus | – | – | – | – | + | – | – | – | – | – | – | – |
| **Gastrointestinal** |  |  |  |  |  |  |  |  |  |  |  |  |
| Hepatomegaly | – | + | + | – | + | + | + | – | + | + | + | – |
| **Failure to thrive** | – | – | + | + | + | + | + | + | + | + | + | – |
| **Endocrine features** | ­– | + | + | – | + | + | + | n.a. | n.a. | + | + |  |
| **Renal features** |  |  |  |  |  |  |  |  |  |  |  |  |
| Proteinuria | – | + | – | – | – | – | – | – | – | – | + | – |
| Tubulopathy | – | – | – | – | – | – | – | – | – | – | – | – |
| **Cardiac features** |  |  |  |  |  |  |  |  |  |  |  |  |
| Pericardial effusion | – | – | – | – | + | + | – | + | + | + | + | – |
| Cardiomiopathy | – | – | hypertrophic | – | – | hypertrophic | + | hypertrophic | + | – | – | – |
| **Skin features** | – | – | – | – | – | – | – | – | – | – | – | – |
| **Inverted nipples** | – | – | + | + | + | + | – | + | + | + | + | – |
| **Abnormal fat distribution** |  |  |  |  | + | + |  |  |  | + | + | – |
| **Laboratory results** |  |  |  |  |  |  |  |  |  |  |  |  |
| Prolonged INR | – | – | + | – | – | – | + | – | + | + | + | – |
| Elevated serum transaminases | – | + | + | – | + | + | + | – | + | + | + | – |
| Low protein C | – | n.a. | + | – | – | n.a. | + | – | + | n.a. | + | – |
| Low protein S | – | n.a. | + | – | + | n.a. | + | – | + | n.a. | + | – |
| Low anithrombin | – | n.a. | + | – | n.a. | n.a. | + | – | + | + | + | – |
| **Follow-up** | | | | | | | | | | | |  |
|  | Improvement of motor skills, epilepsy well controlled by medication, normal liver volume, normal serum transaminases | Improvement of motor skills, epilepsy controlled by medication, Proteinuria since 8m, renal biopsy revealed the congenital nephrotic syndrome | Failure to thrive, subtle improvement of motor skills | Intellectual disability from mild (7y) to moderate (17y), epilepsy since 15y, well controlled by medication | Improvement of motor skills, stroke-like episode at 6y,  Normal serum transaminaes | Pancytopenia, progressive course | Mechanical ventilation since 2nd week of life;  Post mortem examination – liver fibrosis with cholestasis, hypertrophy of the left ventricle | No follow-up | Thrombotic event at 2m, pleural-pericardial window at 9m,  Epileptic seizures from 10m,  Drug-resistant epilepsy diagnosed at 12y | Improvement of motor skills, Feeding by nasogastric tube, enlarged liver volume, Elevated serum transaminases | Feeding by nasogastric tube, several episodes of pericardiocentesis, enlarged licer volume, elevated serum transaminases, coagulopathy, thrombocytopenia, proteinuria | Epilepsy since 2y, peripheral neuropathy, profound intellectual disability (17y), wheel-chair dependent |

| Patient | 13 | 14 | 15 | 16 | 17 | 18 | 19 | 20 | 21 | 22 | 23 |
| --- | --- | --- | --- | --- | --- | --- | --- | --- | --- | --- | --- |
| Gender | F | F | F | M | M | F | F | F | M | M | F |
| Age of onset | 4m | 2m | 4m | Neonatal period | Neonatal period | Neonatal period | 4m | 2y | 12m | 12m | Neonatal period |
| Age at diagnosis | 4y | 2y | 5y | 3m | 4m | 10m | 7m | 2y | 12m | 12m | 10y |
| Diagnosis | ALG13 | ALG13 | ALG13 | ALG1 | ALG1 | ALG1 | ALG3 | MPI | MPI | MPI | PGM1 |
| Molecular analysis | c.320A>G,  p.N107S,  *de novo* | c.320A>G,  p.N107S,  *de novo* | n.a. | c.773C>T,  p.S258L/  c.1182C>G,  p.F394L | c.773C>T,  p.S258L/  c.1182C>G,  p.F394L | Chromosome 16p13.3 deletion involving *ALG1* gene | n.a. | c.1193T>C,  p. 398TI/  c.1193T>C,  p. 398TI | c.1193T>C,  p. 398TI/  c.1193T>C,  p. 398TI | c.656G>A,  p.R219Q/  c.748G>A,  p.G250S | c.988G>C,  p.G330R/  c.1129G>A,  p.E377K |
| Age at last follow-up | 4y | 2y | 6y | 5m | 5m | 10m | 10y 5m | 21y | 14y 5m | 4y | 19y |
| Outcome | Alive | Alive | Alive | Died | Died | Alive | Alive | Alive | Alive | Alive | Died |
| **Neurological** |  |  |  |  |  |  |  |  |  |  |  |
| Muscle hipotonia | + | + | + | – | + | + | + | – | – | – | – |
| Motor retardation | + | + | + | + | + | + | + | – | – | + | – |
| Cerebellar ataxia with cerebellar hypoplasia in brain MRI | – | – | – | – | – | – | – | – | – | – | – |
| Other brain MRI findings | – | Brain atrophy | – | n.a. | n.a. | CT – brain edema | – | – | – | – | n.a. |
| Seizures | + | + | + | + | – | + | – | – | – | – | – |
| Microcephaly | + | + | – | + | + | + | – | – | – | – | – |
| **Hearing impairment** | – | + | – | n.a. | n.a. | n.a. | – | – | – | – | – |
| **Ocular manifestations** |  |  |  |  |  |  |  |  |  |  |  |
| Visual impairment | + | + | + | – | ­– | – | – | – | – | – | – |
| strabismus | – | – | + | – | – | – | – | – | – | – | – |
| Optic nerve hypoplasia/atrophy | – | – | – | – | – | – | – | – | – | – | – |
| **Gastrointestinal** |  |  |  |  |  |  |  |  |  |  |  |
| Chronic diarrhea | – | – | – | – | – | – | – | – | – | + | – |
| Hepatomegaly | – | – | – | + | – | – | – | + | + | + | + |
| **Failure to thrive** | – | – | – | + | + | + | + | – | – | – | + |
| **Endocrine features** | ­– | – | – | – | – | ­– | – | – | – | – | + |
| **Renal features** |  |  |  |  |  |  |  |  |  |  |  |
| Proteinuria | – | – | – | – | – | – | – | – | – | – | – |
| Tubulopathy | – | – | – | – | – | – | – | – | – | – | – |
| **Cardiac features** |  |  |  |  |  |  |  |  |  |  |  |
| Pericardial effusion | – | – | – | – | – | – | – | – | – | – | + |
| Cardiomyopathy | – | – | – | – | – | – | – | – | – | – | +  cardiac insufficiency |
| **Skin features** | – | – | – | – | – | – | – | – | – | – | – |
| **Laboratory results** |  |  |  |  |  |  |  |  |  |  |  |
| Prolonged INR | – | – | – | + | – | – | – | – | – | – | – |
| Elevated serum transaminases | – | – | – | + | – | – | – | + | + | + | + |
| Low protein C | – | – | – | + | + | + | + | + | + | + | n.a. |
| Low protein S | – | – | – | + | + | + | + | + | + | + | n.a. |
| Low anithrombin | – | – | – | + | + | + | + | + | + | + | + |
| Follow-up | | | | | | | | | | | |
|  | Drug-resistant epilepsy,  Severe mental retardation (4y), subtle improvement of motor skills, walks with help (4y), microcephaly | Drug-resistant epilepsy, improvement of motor skills, walks independently (2y), speeks few words, microcephaly | Drug-resistant epilepsy | Spastic tetraparesis, two trombotic events, profound deficiency of antithrombin, protein C and S | Mechanicalm ventillation since 4m of age | Failure to thrive, microcephaly, profound hypotonia | Hypotonia, cachexia, brain atrophy on brain MRI, normal serum transaminases | Mannose treatment since diagnosis, at 21y presenting with normal intellectual development, normal liver volume, normal serum transaminases | Mannose treatment since diagnosis, at 14y 5m presenting with normal intellectual development, normal liver volume, normal serum transaminases | Mannose treatment since diagnosis, at 4y presenting with normal psychomotor development, normal liver volume, normal serum transaminases, and no diarrhea | Progressive cardiac insufficviency since 4y, dilated cardiomiopathy diagnosed at 9y, elevated serum transaminases since 4y, liver biopsy at 10y with results of liver steatosis, start treatment from 16y |

| Patient | 24 | 25 | 26 | 27 | 28 | 29 | 30 | 31 | 32 |
| --- | --- | --- | --- | --- | --- | --- | --- | --- | --- |
| Gender | F | F | M | M | M | F | M | M | M |
| Age of onset | 4m | 6m | Neonatal period | Neonatal period | Neonatal period | Neonatal period | infancy | Prenatally (non-immune hydrops fetalis) | infancy |
| Age at diagnosis | 6m | 6m | 4m | 1y1m | 6m | 1y9m | 9y | 9y, Family screening | 30y, Family screening |
| Diagnosis | SRD5A3 | SRD5A3 | SRD5A3 | SRD5A3 | DPAGT1 | ATP6V0A2 | ATP6AP1 | ATP6AP1 | ATP6AP1 |
| Molecular analysis | c.292_293del,  p.Leu98ValfsX121/  c.292_293del,  p.Leu98ValfsX121 | c.292_293del,  p.Leu98ValfsX121/  c.292_293del,  p.Leu98ValfsX121 | c.424C>T,  p.R142X/  c.424C>T,  p.R142X | c.424C>T,  p.R142X/  c.489C>A,  p.T163X | n.a. | n.a. | c.1284G>A,  p.M428I/  c.1284G>A,  p.M428I | c.1284G>A,  p.M428I/  c.1284G>A,  p.M428I | c.1284G>A,  p.M428I/  c.1284G>A,  p.M428I |
| Age at last follow-up | 4y 4m | 7y 8m | 9y | 1y 5m | 8m | n.a. | 25y | 18y | 36y |
| Outcome | Alive | Alive | Alive | Alive | Died | n.a. | Alive | Alive | Alive |
| **Presentation at diagnosis** | | | | | | | | | |
| **Neurological** |  |  |  |  |  |  |  |  |  |
| Muscle hipotonia | + | + | – | – | + | – | – | – | – |
| Motor retardation | + | + | + | + | + | – | – | – | – |
| Cerebellar ataxia with cerebellar hypoplasia in brain MRI | + | + | + | + | – | ­– | ­– | ­– | ­– |
| Other brain MRI findings | + | + | + | – | Hypomyelination, brain atrophy | pachygyria | – | – | – |
| Seizures | – | – | – | – | – | – | – | – | – |
| Microcephaly | – | – | – | – | – | + | – | – | – |
| **Hearing impairment** | – | – | – | – |  | – | + | + | + |
| **Ocular manifestations** |  |  |  |  | Bilateral cataracts |  |  |  |  |
| Visual impairment | + | + | + | – | – | – | – | – | – |
| strabismus | – | – | – | – | – | – | – | – | – |
| nystagmus | + | + | + | + | – | – | – | – | – |
| Optic nerve hypoplasia/atrophy | + | – | – | – | ­– | ­– | ­– | ­– | ­– |
| **Gastrointestinal** |  |  |  |  |  |  |  |  |  |
| Recurrent vomiting/diarrhoea | – | – | – | – | – | – | – | – | – |
| Hepatomegaly | – | – | – | – | – | – | – | – | – |
| **Failure to thrive** | – | – | – | + | – | – | ­– | ­– | ­– |
| **Endocrine features** | ­– | – | – | – | ­– | – | – | – | – |
| **Renal features** |  |  |  |  |  |  |  |  |  |
| Proteinuria | – | – | – | – | – | – | – | – | – |
| Tubulopathy | – | – | – | – | – | – | – | – | – |
| **Cardiac features** |  |  |  |  |  |  |  |  |  |
| Pericardial effusion | – | – | – | – | ­– | – | – | – | – |
| Cardiomiopathy | – | – | – | – | – |  | – | – | – |
| **Skin features** | – | – | – | – | – | cutis laxa | – | – | – |
| **Laboratory results** |  |  |  |  |  |  |  |  |  |
| Prolonged INR | – | – | – | + | – | – | – | – | – |
| Elevated serum transaminases | + | + | + | + | + | – | + | + | + |
| Low protein C | + | + | + | + | n.a. | + | n.a. | n.a. | n.a. |
| Low protein S | + | + | + | + | n.a. | + | n.a. | n.a. | n.a. |
| Low anithrombin | + | + | + | + | n.a. | + | n.a. | n.a. | n.a. |
| **Follow-up** | | | | | | | | | |
|  | Improvement in motor skills, walks independently,  Normal serum transaminases | Improvement in motor skills, walks independently but abnormal gait pattern (ataxia), says few words, normal serum transaminases | Feeding by gastrostomy (implemented at 6m), severe intellectual disability (9y), tetraparesis | Says few words at 8-9y and few sentences at 10-11y  Moderate intellectual disability (11y), walks independently from 9y of age but abnormal gait pattern (ataxia),  Normal serum transaminases | Mechanical ventillation since 6m | No follow-up | Sensorineural hearing loss (since 8y) requiring cochlear implants, progressive hair loss since 15y, total alopecia and dark (chestnut) skin at 25y,  Normal liver volume, normal serum transaminases, normal urine analysis | Sensorineural hearing loss (since 6y) requiring cochlear implants, at 18y of age presenting with progressive hair loss, leukopenia, mild elevation of serum transaminases, hypogammaglobulinemia,  and glomerular proteinuria | Sensorineural hearing loss from childhood requiring cochlear implants, at 36y of age presenting with total alopecia, dark (chestnut) skin, leukopenia, mild elevation of serum transaminases,  and glomerular proteinuria |
