# Supplementary material for: Clinical, biochemical and molecular phenotype of congenital disorders of glycosylation: long-term follow-up
Source: Orphanet J Rare Dis. 2021 Jan 6;16:17. doi: 10.1186/s13023-020-01657-5 (PMC7789416; doi:10.1186/s13023-020-01657-5)
Supplement: Supplementary file 3 — Additional file 3. Supplementary Table S2. Pearson’s correlation coefficients for % isoforms for the whole cohort of patients (A), PMM2-CDG patients (B), and non-PMM2-CDG patients (C). Correlation values |r|>0.7 marked with colour, the intensity of the hue reflecting the strength of the correlation. [file 13023_2020_1657_MOESM3_ESM.doc]

Table Z

| A | Asiao- | Monosialo- | Disialo- | Trisialo- | Tetrasialo- | Pentasialo- | Heksasialo- |
| --- | --- | --- | --- | --- | --- | --- | --- |
| Asiao- | 1 | 0,447847 | 0,640871 | -0,52112 | -0,85529 | -0,65419 | -0,20206 |
| Monosialo- |  | 1 | 0,230483 | -0,16668 | -0,49676 | -0,37054 | -0,04772 |
| Disialo- |  |  | 1 | -0,4376 | -0,80482 | -0,7663 | -0,36403 |
| Trisialo- |  |  |  | 1 | 0,262683 | 0,008233 | -0,17141 |
| Tetrasialo- |  |  |  |  | 1 | 0,77847 | 0,198376 |
| Pentasialo- |  |  |  |  |  | 1 | 0,536565 |
| Hexasialo- |  |  |  |  |  |  | 1 |

| B | Asiao- | Monosialo- | Disialo- | Trisialo- | Tetrasialo- | Pentasialo- | Heksasialo- |
| --- | --- | --- | --- | --- | --- | --- | --- |
| Asiao- | 1 | 0,672755 | 0,642791 | -0,49707 | -0,82465 | -0,83963 | -0,53543 |
| Monosialo- |  | 1 | 0,425794 | -0,09801 | -0,65875 | -0,63311 | -0,23217 |
| Disialo- |  |  | 1 | -0,10587 | -0,91137 | -0,8544 | -0,7296 |
| Trisialo- |  |  |  | 1 | 0,087538 | 0,177861 | 0,181705 |
| Tetrasialo- |  |  |  |  | 1 | 0,89102 | 0,583147 |
| Pentasialo- |  |  |  |  |  | 1 | 0,824373 |
| Hexasialo- |  |  |  |  |  |  | 1 |

| C | Asiao- | Monosialo- | Disialo- | Trisialo- | Tetrasialo- | Pentasialo- | Heksasialo- |
| --- | --- | --- | --- | --- | --- | --- | --- |
| Asiao- | 1 | 0,526325 | 0,554648 | -0,39629 | -0,87679 | -0,73787 | -0,26578 |
| Monosialo- |  | 1 | 0,504972 | -0,22847 | -0,54566 | -0,56565 | -0,20682 |
| Disialo- |  |  | 1 | -0,24566 | -0,77892 | -0,95091 | -0,45461 |
| Trisialo- |  |  |  | 1 | 0,16167 | 0,295465 | 0,095761 |
| Tetrasialo- |  |  |  |  | 1 | 0,861673 | 0,162902 |
| Pentasialo- |  |  |  |  |  | 1 | 0,449505 |
| Hexasialo- |  |  |  |  |  |  | 1 |

Supplementary Table S2. Pearson’s correlation coefficients for % isoforms for the whole cohort of patients (A), PMM2-CDG patients (B), and non-PMM2-CDG patients (C). Correlation values |r|>0.7 marked with colour, the intensity of the hue reflecting the strength of the correlation.
