# Supplementary material for: Clinical, biochemical and molecular phenotype of congenital disorders of glycosylation: long-term follow-up
Source: Orphanet J Rare Dis. 2021 Jan 6;16:17. doi: 10.1186/s13023-020-01657-5 (PMC7789416; doi:10.1186/s13023-020-01657-5)
Supplement: Supplementary file 4 — Additional file 4. Supplementary Table S3. Comparison of means (with standard deviation) of Tf isoform % for the whole cohort of patients, PMM2-CDG patients and non-PMM2-CDG patients. The last column reports the p-values of the tests comparing the sample means (t-test or Welsh test). [file 13023_2020_1657_MOESM4_ESM.doc]

|  | Mean Tf isoform % for the whole cohort (SD in parenthesis) | Mean Tf isoform % for PMM2-CDG  (SD in parenthesis) | Mean Tf isoform % for non-PMM2-CDG (SD in parenthesis) | p-value of the test assessing statistical significance of differences in means between PMM2-CDG and non-PMM2-CDG |
| --- | --- | --- | --- | --- |
| Asialo- | 10.2 (10.1) | 13.2  (9.3) | 9.8  (10.8) | 0.3852 |
| Monosialo- | 2.2  (2.8) | 2.3  (1.3) | 1,6  (1.7) | 0.2754 |
| Disialo- | 22.8  (11.2) | 27.3  (10.1) | 22.5  (11.7) | 0.269 |
| Trisialo- | 12.5  (7.5) | 9.3  (3.8) | 10.4  (3.3) | 0.4092 |
| Tetrasialo- | 38.9  (12.7) | 36.1  (13.5) | 40.2  (13) | 0.4264 |
| Pentasialo- | 11.1  (5.2) | 9.8  (3.8) | 12.8  (6.6) | 0.07168 |
| Hexasialo- | 2.3  (1.5) | 2.1  (1) | 2.7  (2) | 0.3965 |

Supplementary Table S3. Comparison of means (with standard deviation) of Tf isoform % for the whole cohort of patients, PMM2-CDG patients and non-PMM2-CDG patients. The last column reports the p-values of the tests comparing the sample means (t-test or Welsh test).
