# Supplementary material for: Clinical, biochemical and molecular phenotype of congenital disorders of glycosylation: long-term follow-up
Source: Orphanet J Rare Dis. 2021 Jan 6;16:17. doi: 10.1186/s13023-020-01657-5 (PMC7789416; doi:10.1186/s13023-020-01657-5)
Supplement: Supplementary file 5 — Additional file 5. Supplementary Table S4. Means of % tf isoforms before and during treatment for CDG I/II patient (table A), patient #20 with MPI-CDG-I (table B) and patient #21 with MPI-CDG-I (table C). Comparison of means (A and B) or of mean with a single value (C) was performed with applicable statistical test (either t-test, Welsh test, or one-sample t-test). [file 13023_2020_1657_MOESM5_ESM.doc]

| **A**  isoform | Mean % before start of treatment (7 measurements) | Mean % during treatment (11 measurements) | Reference range | p-value of the test comparing two means |
| --- | --- | --- | --- | --- |
| Asialo | 19.6 | 4.4 | 0 | <0.00001 |
| Monosialo | 14.1 | 5.1 | 0 | <0.00001 |
| Disialo | 19 | 12.2 | 1.5-6.2 | <0.00001 |
| Trisialo | 11.2 | 11.8 | 7.4-17.1 | 0.367 |
| Tetrasialo | 24.3 | 51 | 55.7-66.2 | <0.00001 |
| Pentasialo | 8.4 | 13 | 13.2-19.9 | <0.00001 |
| Hexasialo | 2.2 | 2.5 | 2.5-5.6 | 0.316 |

| **B**  isoform | Mean % before start of treatment (4 measurements) | Mean % during treatment (12 measurements) | Reference range | p-value of the test comparing the mean with the value before treatment |
| --- | --- | --- | --- | --- |
| Asialo | 9.8 | 3 | 0 | 0.07 |
| Monosialo | 1.7 | 1 | 0 | <0.00001 |
| Disialo | 25.7 | 14.8 | 1.5-6.2 | 0.0008 |
| Trisialo | 11.7 | 8.2 | 7.4-17.1 | <0.00001 |
| Tetrasialo | 34.9 | 52.9 | 55.7-66.2 | <0.00001 |
| Pentasialo | 12.5 | 16.4 | 13.2-19.9 | <0.00001 |
| Hexasialo | 4 | 3.6 | 2.5-5.6 | 0.46 |

| **C**  isoform | % value before start of treatment (1 measurement) | Mean % during treatment (11 measurements) | Reference range | p-value of the test comparing the mean with the value before treatment |
| --- | --- | --- | --- | --- |
| Asialo | 12.2 | 1.3 | 0 | <0.00001 |
| Monosialo | 2 | 0.7 | 0 | <0.00001 |
| Disialo | 29 | 14.5 | 1.5-6.2 | <0.00001 |
| Trisialo | 8.3 | 7.7 | 7.4-17.1 | 0.432 |
| Tetrasialo | 30.6 | 56.6 | 55.7-66.2 | <0.00001 |
| Pentasialo | 11.8 | 15.5 | 13.2-19.9 | <0.00001 |
| Hexasialo | 5.2 | 3.4 | 2.5-5.6 | <0.00001 |

Supplementary Table 4 Means of % tf isoforms before and during treatment for CDG I/II patient (table A), patient #20 with MPI-CDG-I (table B) and patient #21 with MPI-CDG-I (table C).
Comparison of means (A and B) or of mean with a single value (C) was performed with applicable statistical test (either t-test, Welsh test, or one-sample t-test).
